# Negative mood reverses devaluation of goal-directed drug-seeking favouring an incentive learning account of drug dependence

**DOI:** 10.1007/s00213-015-3977-z

**Published:** 2015-06-05

**Authors:** Lee Hogarth, Zhimin He, Henry W. Chase, Andy J. Wills, Joseph Troisi, Adam M. Leventhal, Amanda R. Mathew, Brian Hitsman

**Affiliations:** School of Psychology, University of Exeter, Washington Singer Building, Perry Road, Exeter, EX4 4QG UK; Institute of Psychiatry, King’s College London, London, UK; Department of Psychiatry, University of Pittsburgh, Pittsburgh, PA USA; School of Psychology, University of Plymouth, Plymouth, UK; Department of Psychology, Saint Anselm College, Manchester, NH USA; Departments of Preventive Medicine and Psychology, University of Southern California Keck School of Medicine, Los Angeles, CA USA; Department of Psychiatry and Behavioral Sciences, Medical University of South Carolina, Charleston, SC USA; Department of Preventive Medicine, Northwestern University Feinberg School of Medicine, Chicago, IL USA

**Keywords:** Incentive learning, Goal-directed learning, Drug-seeking, Negative mood, Depression, Allostasis, Discriminative stimuli, Motivating operations, Negative reinforcement

## Abstract

**Background:**

Two theories explain how negative mood primes smoking behaviour. The stimulus–response (S-R) account argues that in the negative mood state, smoking is *experienced* as more reinforcing, establishing a direct (automatic) association between the negative mood state and smoking behaviour. By contrast, the incentive learning account argues that in the negative mood state smoking is *expected* to be more reinforcing, which integrates with instrumental knowledge of the response required to produce that outcome.

**Objectives:**

One differential prediction is that whereas the incentive learning account anticipates that negative mood induction could augment a novel tobacco-seeking response in an extinction test, the S-R account could not explain this effect because the extinction test prevents S-R learning by omitting experience of the reinforcer.

**Methods:**

To test this, overnight-deprived daily smokers (*n* = 44) acquired two instrumental responses for tobacco and chocolate points, respectively, before smoking to satiety. Half then received negative mood induction to raise the expected value of tobacco, opposing satiety, whilst the remainder received positive mood induction. Finally, a choice between tobacco and chocolate was measured in extinction to test whether negative mood could augment tobacco choice, opposing satiety, in the absence of direct experience of tobacco reinforcement.

**Results:**

Negative mood induction not only abolished the devaluation of tobacco choice, but participants with a significant increase in negative mood increased their tobacco choice in extinction, despite satiety.

**Conclusions:**

These findings suggest that negative mood augments drug-seeking by raising the expected value of the drug through incentive learning, rather than through automatic S-R control.

## Introduction

A key debate in contemporary addiction theory is whether the transition from recreational drug use to clinical drug dependence is driven by the emergence of automatic control over drug-seeking (Everitt and Robbins 2013; Koob 2013) or supernormal reinforcement value of the drug increasing intentional choice of this commodity (Bickel et al. 2014; Heyman 2013). As clinical drug dependence is comorbid with psychiatric illness (SAMHSA 2012; Swendsen et al. 2010), one might recast this question as whether psychiatric states increase automatic or intentional drug choice. To explore this question, the current study used an outcome-devaluation assay to test whether negative mood would increase goal-directed drug-seeking in an extinction test, consistent with negative mood exerting its effect on drug-seeking through intentional rather than automatic processes (Hogarth 2012; Hutcheson et al. 2001; Willner and Jones 1996). The study should contribute to our understanding of how depression promotes drug dependence (Breslau et al. 1998; Hitsman et al. 2013; Hughes 1999; Kassel et al. 2007) and provide insight into the mechanisms underpinning the transition to drug dependence.

Most accounts of how negative mood promotes tobacco-seeking draw inspiration from the self-medication hypothesis (Khantzian 1997). The core idea is that smoking acutely alleviates negative mood, and this improvement in state back towards homeostasis (Brody et al. 2009; Ramsay and Woods 2014) increases the reinforcement value of smoking (Hursh and Silberberg 2008). Crucially, it is proposed that because negative mood signals that smoking has greater reinforcement value, negative mood acquires the capacity to prime smoking behaviour (Audrain-McGovern et al. 2014; Leventhal et al. 2014b).

Theories differ in their description of the mechanisms by which negative mood comes to acquire control over smoking behaviour. Early accounts described negative mood as an internal instrumental *discriminative stimulus* (*S*^D^) which ‘sets the occasion’ (Skinner 1938) in which smoking behaviour is more reinforcing, and thus comes to prime smoking behaviour, although exactly how was not well defined (Carmody 1989; Pomerleau and Pomerleau 1984).

This type of account was developed by negative reinforcement theories (Baker et al. 2004), allostasis theories (Koob 2009; Koob and Volkow 2010) and incentive habit theories (Belin et al. 2013). These accounts argue that experience of the greater reinforcement value of smoking in the negative mood state establishes a strong direct link between the negative mood state and the motor sequence of smoking. This direct link has been variously described as automatic, unconscious, preconscious, habitual and compulsive. In essence, these theories have adopted Hull’s (Hull 1943) stimulus–response/reinforcement (S-R) account of instrumental discrimination learning, wherein the negative mood state (S) acquires capacity to directly elicit the motor sequence of smoking (R) without retrieving an expectation of the reinforcer produced by that response. Such S-R accounts are attractive because they can explain how negative mood could prime smoking behaviour automatically, bypassing the individual’s intentions or beliefs about the nature of the drug and its current value.

Behaviour analysts have questioned S-R accounts of negative mood on the logical grounds that topographically different smoking behaviours are required to produce reinforcement in different contexts (for instance, obtaining cigarettes from a shop versus a machine requires different sequences). Negative mood is not sufficiently discriminating on its own, they argue, to elicit the appropriate response sequence in each context via S-R mapping, because negative mood is largely the same irrespective of context (Dougher and Hackbert 2000; Laraway et al. 2014; Michael 1993; Troisi 2014). For this reason, the behaviour analysts have argued that negative mood is more accurately described as a *motivating operation* (MO), rather than a *S*^D^, because it predicts the greater reinforcement value of smoking irrespective of the specific response that is required to produce that reinforcer in the context. *S*^D^s by contrast, are typically external cues which are scheduled to predict that a particular response will produce an outcome. Consequently, they argue, the motivating operation, negative mood, should come to lower the threshold enabling external discriminative stimuli to evoke the specific response that produces the valued outcome in the context. On this view, negative mood does not elicit smoking directly (as predicted by S-R theory), but rather, modulates the ability of external discriminative stimuli to evoke smoking.

Incentive learning theory has taken this type of modulatory account further (Balleine et al. 1994; Dickinson and Balleine 2010; Heyes and Dickinson 1990; see also, Pittenger and Bevins 2013). On this view, negative mood functions as an internal *motivational state*, which is much like a MO in reliably predicting that smoking has greater reinforcement value. However, rather than automatically modulating the efficacy of external *S*^D^s, incentive learning theory argues that smokers learn from experience that in the negative mood state, smoking is more reinforcing. This incentive learning experience enables the motivational state, negative mood, to subsequently retrieve an expectation that smoking currently has a higher value, which manifests as subjective desire for that outcome. This outcome expectancy is integrated with goal-directed instrumental knowledge of which response produces that outcome in the external discriminative context (Bradfield and Balleine 2013; Trask and Bouton 2014), and this conjunction determines the selection and performance of the appropriate smoking response. Thus, incentive learning argues that negative mood primes smoking behaviour via a conjunction of explicit desire and instrumental belief, whereas the S-R account proposes that negative mood primes smoking behaviour directly without intervening decision processes.

The incentive learning, MO and S-R accounts fall on a continuum with the incentive learning and S-R accounts at each extreme, so only the differential predictions of these latter two positions will be explored in the remainder of the paper. The incentive learning and S-R accounts make a differential prediction as to whether the increase in self-report desire to smoke associated with negative mood is causal or epiphenomenal in driving smoking behaviour. Existing experimental data cannot readily distinguish these two positions because although desire and behaviour are commonly correlated, causal control by desire over behaviour has not been empirically confirmed. To be specific, studies have shown that smokers who are prone to negative mood or are depressed verbally report intentionally smoking in order to alleviate negative mood (Lerman et al. 1996), and more often report smoking as their most preferred activity (Audrain-McGovern et al. 2014; Spring et al. 2003), show quicker latency to smoke and greater willingness to pay for cigarettes (Leventhal et al. 2014b) and show preferential selection of smoking over money reinforcement in a concurrent choice progressive ratio schedule (Audrain-McGovern et al. 2014). Negative mood induction is associated with similar enhancements of smoking motivation. Negative mood induction increases self-reported desire to smoke (Brandon et al. 1996; Payne et al. 1991; Perkins et al. 2013; Vinci et al. 2012), increases smoking behaviour (Conklin and Perkins 2005; Fucito et al. 2010; Payne et al. 1991) and increases response rate in an instrumental progressive ratio task reinforced by cigarette puffs (Willner and Jones 1996). Collectively, these studies show that negative mood (trait or state) produces corresponding increases in a self-reported desire to smoke, instrumental tobacco-seeking and smoking behaviour. However, it remains unclear whether self-reported desire is causal or epiphenomenal in driving smoking-related behaviour. Thus, the empirical co-occurrence of desires and smoking behaviours under negative mood, whilst suggestive, does not completely resolve the issues of whether negative mood controls smoking behaviour effect via incentive learning or S-R mechanisms.

Fortunately, the incentive learning and S-R accounts make a second differential prediction that is more tractable to experimental dissection—whether or not negative mood induction could augment a novel tobacco-seeking response in an extinction test. The importance of the extinction test is that it prevents the opportunity for direct *experience* of the greater value of tobacco outcome in the negative mood state strengthening the S-R association between the mood state and the tobacco-seeking response. Thus, S-R theory predicts that negative mood induction should not enhance a novel tobacco-seeking response in an extinction test and could not readily explain this effect if it was found. By contrast, the extinction test does allow an *expectation* about the current high value of tobacco evoked by negative mood to integrate with knowledge of the response that produces that outcome, to augment performance of the response. Consequently, the finding that negative mood induction enhances a novel tobacco-seeking response in extinction would support the incentive learning account and disconfirm the S-R account of how negative mood primes smoking behaviour.

The extinction test has become a standard procedure for testing whether naturally reinforced responses (Balleine et al. 1994; Kosaki and Dickinson 2010) or drug-seeking responses (Corbit et al. 2012; Dickinson et al. 2002; Hutcheson et al. 2001; Miles et al. 2003) are governed by incentive or S-R learning in animals. In one key study, Hutcheson et al. (2001) found that in animals that had experienced the greater value of heroin in withdrawal, being shifted to a state of heroin withdrawal augmented performance of a heroin-seeking response in extinction, even though that response had never before been reinforced in the withdrawal state. Thus, withdrawal must have augmented heroin-seeking by retrieving an expectation of the greater reinforcement value of heroin, which integrated with goal-directed knowledge of the response-heroin contingency, to augment performance of that response. This increase in heroin-seeking could not have been driven by the strengthening of any S-R association controlling the response because the extinction test prevented direct experience of the greater reinforcement value of heroin in the withdrawal state modulating the propensity to make the response through S-R learning.

Similar to Hutcheson et al. (2001), the current human procedure tested whether negative mood would function as a motivational state raising the expected value of the drug in the extinction test. In previous versions of the current human design (Hogarth 2012; Hogarth and Chase 2011; Hogarth et al. 2013), smokers first underwent concurrent choice training in which two key press responses earned points notionally exchangeable for tobacco and chocolate rewards, respectively, and participants reported explicit knowledge of these instrumental response-outcome (R-O) contingencies. Smoking to satiety was then used to decrease the expected value of the tobacco outcome. Finally, choice between the two responses was tested in nominal extinction, where participants believed that the responses continued to earn their outcomes, but these outcomes were not displayed until the end (to prevent direct experience of the outcomes modifying the tendency to make each response in the extinction test through S-R learning). These studies found that the devaluation treatment (smoking satiety) reduced tobacco choice in the extinction test relative to concurrent training. Thus, the tobacco-seeking response in the extinction test was demonstrably governed by knowledge of the current expected low value of the tobacco outcome in the sated state, integrated with knowledge of the response-outcome contingencies learned in the concurrent training phase. This effect could not be explained by S-R learning, because the extinction test prevented direct experience of the current low value of the tobacco outcome weakening the S-R association controlling the response.

The question at stake in the present experiment was whether negative mood induction (compared with positive mood induction) administered immediately prior to the extinction test would reverse/oppose the devaluation of goal-directed tobacco-seeking produced by satiety, consistent with negative mood functioning as a motivational state raising the expected value of the drug. Numerous studies have shown that negative mood induction increases drug motivation, but it is not clear if this effect can compete with primary motivational states. By testing whether negative mood can counter drug satiety, we can ascertain whether negative mood is a strong motivational state, capable of competing with primary motivational states. An important precedent for this ‘oppositional’ design comes from Willner and Jones (1996). They found that smoking to satiety reduced response rate on the progressive ratio schedule in which cigarette puffs served as the reinforcer (also consistent with Perkins et al. 1994; Perkins et al. 1997). Crucially, negative mood induction partially reversed the devaluation effect produced by satiety, raising response rates back towards the abstinent baseline. Although negative mood opposed satiety, it is not clear how, given that tobacco-seeking was reinforced with puffs at test. Negative mood may have augmented tobacco-seeking by raising the expected value of tobacco or by enhancing the experience of puffing reinforcement which strengthened the S-R association controlling the response. The key innovation in the present study was to test whether negative mood induction would reverse the devaluation of tobacco-seeking in an extinction test. This effect would favour the incentive learning over the S-R account of how negative mood primes drug-seeking behaviour. More broadly, if mood functions as a motivational state, opposing satiety, this would extend the scope of incentive learning theory to include not just primary states like hunger, thirst, cold, sexual arousal etc. but to also include ‘higher-level’ psychiatric emotional states, like sadness, regret, confusion, anxiety etc., providing principled insight into how these emotional states modulate goal-directed behaviour (Dickinson and Balleine 2010).

## Method

### Participants

Participants were daily smokers (*n* = 48; 50 % male) recruited from students and staff at the University of Nottingham. All were asked to abstain from smoking for at least 3 h prior to the study, which amounted to overnight abstinence for the majority. In order to increase compliance with abstinence procedures, participants were informed that abstinence would be confirmed with a breath CO measurement at the start of the experiment (although this was not used as an exclusion criterion). Exclusion criteria employed during initial e-mail vetting were current use of illicit drugs except cannabis; aged less than 18 or over 40 years of age; significant current or past medical or psychiatric illness (including mood disorder); poor physical health; allergies; or dietary sensitivities to chocolate. Testing was between 10 a.m. and 5 p.m., lasting 1 h/subject. During initial contact, participants were told that they would be paid £10 for participation plus the additional chocolate and cigarettes won in a computer task up to the value of £5. This was a deception. At the end, during debrief, participants were asked if they would be happy to receive £5 extra (£15 in total), ‘to save the experimenter having to restock the items’. All participants accepted this. This arrangement ensures that participants believe they are earning tobacco and chocolate during the instrumental task, and mitigates the ethical problem of paying participants in cigarettes. Informed consent was obtained in accordance with the declaration of Helsinki, and ethical approval was granted by the University of Nottingham, School of Psychology Ethics Committee.

### Procedure

#### Breath CO—baseline

After informed consent was obtained, participants were presented with a Bedfont Smokerlyzer and told that this device measures the time of their last cigarette. Participants self-reported their time of last cigarette (none reported smoking within 3 h, consistent with instructions) before providing their ‘baseline’ exhaled CO reading.

#### Questionnaires

A questionnaire pack followed which included smoking history (age, cigarettes per day, years smoking, age of onset), the Cigarette Dependence Scale (CDS-5; Etter et al. 2003) and the Questionnaire of Smoking Urges (QSU; Tiffany and Drobes 1991).

#### Reward desire—baseline

Participants were then shown the rewards on the table: A packet of ten cigarettes of their preferred brand and a 200-g bar of Cadbury dairy milk (both sealed) above the keyboard on the same side as the key that earned that outcome in the task that followed. Their baseline desire for these rewards was recorded with two questions: ‘To what extent do you agree with the following statements? I would like to (smoke a cigarette right now/eat chocolate right now)’ with a 7-point Likert scale underneath ranging from ‘strongly disagree’ to ‘strongly agree’.

#### Concurrent choice—baseline

The aim of concurrent training was to establish two instrumental responses (key presses), which earn distinct reward. Participants were faced with the physical rewards (a pack of ten cigarettes of their preferred brand and a 200-g bar of Cadbury dairy milk) and on-screen instructions which stated: ‘This is a game in which you can win the cigarettes and chocolate in front of you. In each trial, press the D or H key to see if you have won a point for these rewards. You will only win on some trials. Press the space bar to begin’. Each trial began with the centrally presented fixation cross, which remained until either the D or H key was pressed. Pressing one key immediately presented the outcome text ‘tobacco point’, whereas pressing the other key produced the outcome text ‘chocolate point’, for 1 s. The response-outcome assignment was counterbalanced between participants. Each key had only a 50 % chance of yielding its respective outcome. On non-rewarded trials, the outcome text ‘nothing’ was presented. A random inter-trial interval between 750 and 1250 ms interceded between outcome offset and the fixation cross of the next trial. There were 40 trials of concurrent training in total. Per cent choice of the tobacco over the chocolate response was the main measure, where 50 % = indifference, >50 % = tobacco preference and < 50 % = chocolate preference.

#### Contingency knowledge test

Immediately following concurrent training, participants were tested for knowledge of the instrumental contingencies through the on-screen questions: ‘Which key earned tobacco/chocolate, the D or the H key? Please choose carefully’. The order of the two questions was randomised. Participants who got either question wrong were excluded (*n* = 4).

#### Devaluation by smoking satiety

All participants were then told: ‘We want to test how much you like smoking. There will be a 10-min break in which you can smoke as much or as little as you wish of a cigarette. Please report the pleasantness of each puff you take on this smoking satiety questionnaire’. On this questionnaire, participants reported the pleasantness of each puff they consumed on visual analogue scale, enabling quantification of the number of puffs consumed and the decline in pleasure rating from the first to the last puff. Participants were escorted outside the building to a smoking area and left alone during the smoking period to minimise disturbance. The experimenter stated that they would return when the time was up to facilitate ad libitum consumption. The experimenter returned in 10 min.

#### Reward desire and breath CO - post devaluation

The impact of devaluation on tobacco and chocolate desire was tested immediately after smoking satiety by participants completing the desire questions again (as above). Breath CO was recorded for a second time to quantify smoke exposure.

#### Mood state—baseline

To determine participants’ baseline mood, they were presented with the on-screen question ‘How do you currently feel?’ with a 9-point Likert scale ranging from ‘Happy’ at 1, ‘Neutral’ at 5 and ‘Sad’ at 9.

#### Mood induction procedure

A Velten mood induction procedure was then employed derived from previous studies (Berna et al. 2010; Lenton and Martin 1991; Richell and Anderson 2004; Velten 1968). Participants were presented with the instructions ‘You will now be shown a series of statements that represent a particular type of mood. Read each of the statements to yourself and focus your attention on it. Your success at coming to experience this mood will largely depend on your willingness to accept and respond to the idea in each statement and to allow each statement to act upon you. Attempt to respond to the feeling suggested by each statement. Then try to think of yourself as moving into that state. If it is natural for you to do so, try to visualise a scene in which you have had such a feeling. Press the space bar to begin’. Instructions were followed by 16 statements presented for 10 s each in random order, separated by a random ITI of 2500 to 3500 ms. The two randomly assigned groups (positive and negative) were exposed to different statements. The negative statements were: *I feel a little down today*; *My work is harder than I expected*; *Sometimes I feel so guilty that I can*’*t sleep*; *I wish I could be myself*, *but nobody likes me when I am*; *Today is one of those days when everything I do is wrong*; *I doubt that I*’*ll ever make a contribution in the world*; *I feel like my life is in a rut that I*’*m never going to get out*; *My mistakes haunt me*, *I*’*ve made too many*; *Life is such a heavy burden*; *I*’*m tired of trying*; *Even when I give my best effort*, *it just doesn*’*t seem to be good enough*; *I don*’*t think things are ever going to get better*; *I feel worthless*; *What*’*s the point of trying*; *I feel cheated by life*; *Every time I turn around*, *something else has gone wrong*. The positive statements were: *I feel cheerful and lively*; *On the whole*, *I have very little difficulty in thinking clearly*; *I*’*m pleased that most people are so friendly to me*; *I can make friends extremely easily*; *I feel enthusiastic and confident now*; *There should be a lot of good times coming along*; *I*’*m able to do things accurately and efficiently*; *I know that I can achieve the goals I set*; *I have a sense of power and vigour*; *I*’*m feeling amazingly good today*; *I feel highly perceptive and refreshed*; *I can concentrate hard on anything I do*; *My thinking is clear and rapid*; *Life is so much fun*; *It seems to offer so many sources of fulfilment*; *Life is firmly in my control*; *I*’*m really feeling sharp now*. Both groups listened to music during these statements played through noise cancelling headphones. The negative group received Barber’s Adagio for Strings, whereas the positive group received Mozart’s Einekleine Nachtmusik (Morrison and O’Connor 2008). A positive and negative mood group were contrasted in an attempt to match the arousing effects of the two mood conditions, whilst manipulating the valence. A neutral group was not included because the contrast of the two more extreme groups offered the best strategy to detect an effect.

#### Mood state—post-mood induction

To measure participants’ mood following mood induction, they were again presented with the on-screen question ‘How do you currently feel?’ with a 9-point Likert scale ranging from ‘Happy’ at 1, ‘Neutral’ at 5 and ‘Sad’ at 9. The two groups were expected to diverge in their mood compared with their pre-induction baseline.

#### Extinction test

Finally, participants completed the concurrent choice procedure again, in extinction, to evaluate the combined impact of satiety and mood induction on tobacco-seeking. Participants were presented with the on-screen instructions: ‘You can now earn cigarettes and chocolate by pressing the D or H keys as before. You will only be told how many of each reward you have earned at the end of the experiment. You will also be asked to examine the mood statements. Press the space bar to begin’. The extinction test was identical to concurrent training except that outcomes are omitted from trials. Thus, the initial fixation cross appeared until a response choice was made, which immediately launched the ITI before to the next trial. In addition however, to ensure that the mood induction continued throughout this test phase, one of the mood statements (randomly selected from the set of 16 for the group) was presented for 3 s prior to the fixation cross of each choice trial, and remained on until a response choice was made. The per cent choice of the tobacco over the chocolate key was the main measure. The question at stake was whether satiety and mood induction would modulate the proportion of tobacco choices in this extinction test relative to the baseline concurrent choice phase.

## Results

### Participants

Four participants were excluded due to reporting incorrect knowledge of the response-outcome contingencies after the baseline concurrent choice stage, leaving 20 negative and 24 positive participants. Overall, participants were 23.8 years (std = 4.2; range = 19–34) of age, smoked 9.5 (5.7; 2–23) cigarettes/day, had smoked for 6.2 years (4.8; 1–21), started at 15.9 years (2.6; 11–26) of age, had a CDS-5 score of 15.0 (4.0; 5–22) out of a maximum score of 25 and a QSU score of 4.2 (1.4; 1–7). There were no significant difference between the negative and positive group with respect to these characteristics, *ts*(42) < −1.28, *ps* > 0.20, and there was also no group difference in gender ratio, *X*^2^(1) = 0.1, *p* = 0.74.

### Manipulation effects on self-report

Table 1 shows the effect of smoking satiety and mood induction on self-report measures and breath CO. The groups did not differ in their experience of smoking satiety but did diverge with respect to mood following mood induction. Specifically, analysis of variance (ANOVA) with the breath CO data yielded a main effect of time (baseline, post-devaluation), *F*(1.42) = 45.42, *p* < 0.001, *ŋ*_p_^2^ = 0.52, demonstrating that smoking took place during the satiety manipulation, and there was no main effect of group or interaction between time and group, *F* < 1. The groups also showed no difference in the number of puffs consumed, *F*(1.42) = 1.3, *p* = 0.26. ANOVA with the reward desire data yielded no significant main effects of interactions involving group, *F*(1.42) < 2.62, *p* > 0.11, but revealed a significant interaction between reward (tobacco, chocolate) and time (baseline, post-satiety), *F*(1.42) = 89.98, *p* < 0.001, *ŋ*_p_^2^ = 0.68. This interaction was due to desire for tobacco significantly decreasing after satiety, *F*(1.43) = 145.29, *p* < 0.001, *ŋ*_p_^2^ = 0.77, whereas desire for chocolate showed no significant change, *F*(1.43) = 3.15, *p* = 0.08, *ŋ*_p_^2^ = 0.07. Thus, smoking satiety decreased smoking pleasure and tobacco desire, comparably across groups.

**Table 1:** The effect of smoking satiety (top) and mood induction (bottom two rows) on adjunct measures

Measure	Group	
	Negative	Positive
Breath CO—baseline	3.9 (0.8)	3.8 (0.6)
Breath CO—post-devaluation	5.7 (0.9)	5.7 (0.6)
Number of puffs	17.1 (1.4)	15.3 (1.0)
Pleasure first puff	79.5 (5.1)	83.9 (3.8)
Pleasure last puff	61.0 (5.9)	49.8 (5.9)
Desire tobacco—baseline	5.5 (0.3)	6.0 (0.2)
Desire tobacco—post-satiety	3.0 (0.4)	2.5 (0.3)
Desire chocolate—baseline	3.1 (0.4)	3.2 (0.3)
Desire chocolate—post-satiety	3.6 (0.4)	3.5 (0.4)
Mood state—pre-induction	3.2 (0.4)	3.3 (0.3)
Mood state—post-induction	5.6 (0.4)	2.8 (0.3)

Breath CO is in parts per million. Pleasure of puffs is per cent of visual analogue scale. Desire scores are on a 7-point scale (positive numbers equal greater desire). Mood state scores are on a 9-point scale (positive numbers equal greater sadness) Numbers are mean and sem in brackets

ANOVA with self-reported mood data yielded a significant interaction between time (pre-induction, post-induction) and group, *F*(1.42) = 29.95, *p* < 0.001, *ŋ*_p_^2^ = 0.42, where the negative group reported a significant increase in negative mood, *F*(1.19) = 21.33, *p* < 0.001, *ŋ*_p_^2^ = 0.53, and the positive group showed a significant increase in positive mood, *F*(1.23) = 5.41, *p* = 0.03, *ŋ*_p_^2^ = 0.19. Thus, the two mood induction procedures shifted mood in the expected directions.

### Effects of satiety and mood on tobacco choice

**Fig. 1:**
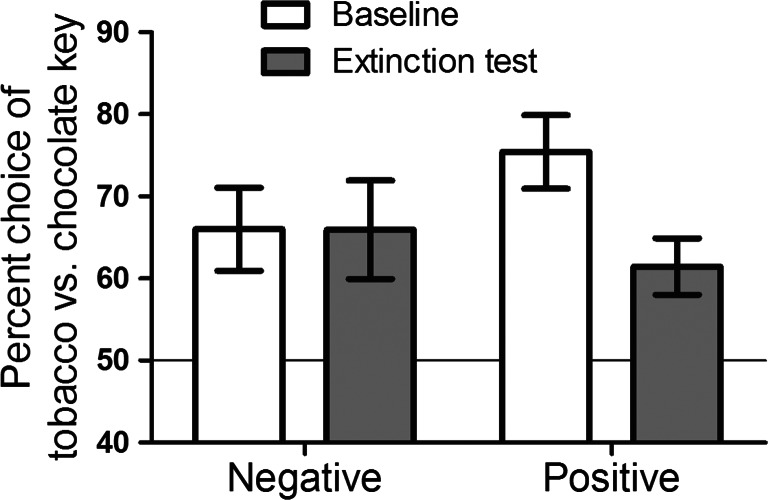
Mean per cent tobacco versus chocolate choice (±SEM) at baseline and extinction test for the negative and positive mood induction group (50 % = indifference; >50 % = tobacco preference; <50 % = chocolate preference). The extinction test was conducted after satiety and mood induction, revealing the combined effect of these variables on goal-directed tobacco-seeking. Satiety reduced goal-directed tobacco-seeking in the positive group, but this effect was abolished by negative mood induction

Baseline concurrent training contained 40 choice trials, and there was a significant linear increase in tobacco choice across successive quarters (62, 69, 65 and 71 %), *F*(1.43) = 6.20, *p* < 0.02, *ŋ*_p_^2^ = 0.13. For this reason, the final quarter (10 trials) of the baseline concurrent choice task were employed as the baseline. Figure 1 shows per cent tobacco choice at this baseline and in the extinction test following satiety and mood induction for the two groups. ANOVA with these data produced a significant interaction between group (negative, positive) and block (baseline, extinction), *F*(1.42) = 5.4, *p* = 0.02, *ŋ*_p_^2^ = 0.11, a significant main effect of block, *F*(1.42) = 5.5, *p* = 0.02, *ŋ*_p_^2^ = 0.12, and no main effect of group, *F* < 1. Furthermore, the main effect of block was significant in the positive group, *F*(1.23) = 12.28, *p* = 0.002, *ŋ*_p_^2^ = 0.35, but not in the negative group, *F* < 1. These data indicate that satiety decreased tobacco choice in the extinction test relative to baseline, consistent with previous findings (Hogarth 2012; Hogarth and Chase 2011; Hogarth et al. 2013). However, this devaluation effect was completely abolished by negative mood induction, suggesting that negative mood functioned as a motivational state raising the expected value of tobacco in opposition to satiety.

### Relationship between the change in self-reported mood and tobacco-seeking

**Fig. 2:**
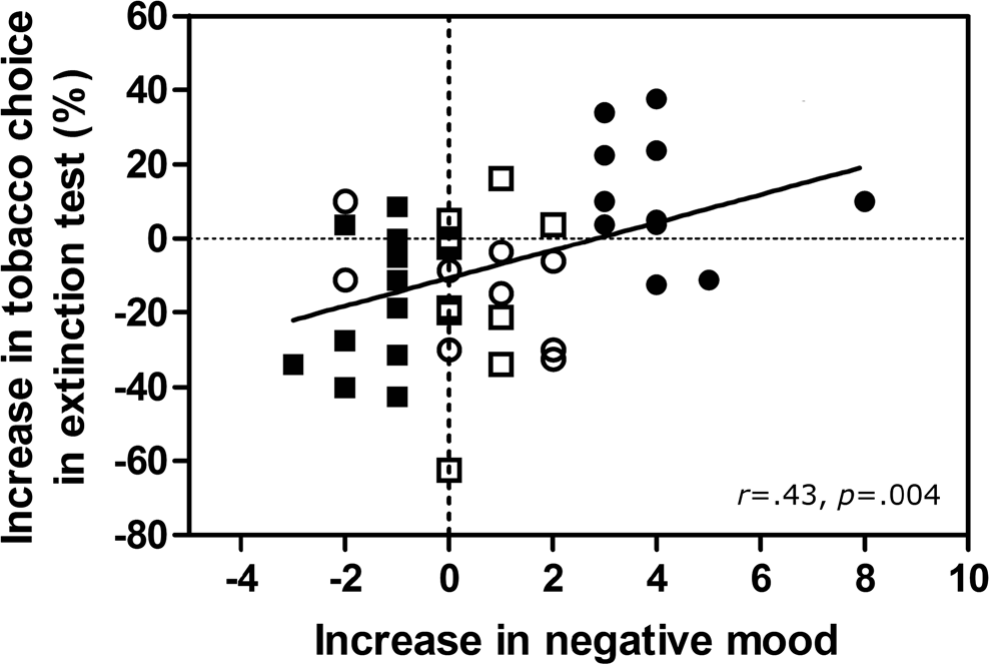
Relationship between the change in self-reported mood (pre- to post-mood induction), with the change in tobacco choice (from baseline to extinction test with satiety mood induction in between). Positive mood values reflect increased sadness, whereas negative values reflect increased happiness. Positive tobacco choice scores reflect increased tobacco choice, whereas negative scores reflect decreased tobacco choice. Symbols identify the four sub-groups employed in Fig. 3: high positive (*filled squares*), low positive (*empty squares*), low negative (*empty circles*) and high negative (*filled circles*) who did and did not show significant changes in mood following induction. The scatterplot shows that increased negative mood was associated with increased tobacco choice at test, opposing satiety

Previous studies have reported correlations between negative subjective reactivity following induction procedures and subsequent increases in drug motivation (e.g., Sinha et al. 2008). This association was examined in Fig. 2, which shows that the change self-reported in mood from pre- to post-mood induction correlated significantly with the change in tobacco-seeking from baseline to the extinction test, *r* = 0.43, *p* = 0.004. This correlation indicates that an increase in self-reported negative mood was associated with an increase in tobacco choice in the extinction test, reversing the effect of satiety, which otherwise decreased tobacco choice. Multiple regression analyses indicated that the *absolute level* of self-reported mood measured pre-induction (*p* = 0.11) or post-inducted (*p* = 0.073) did not predict the change in tobacco choice, but the *change* in mood from pre- to post-induction was the effective predictor (*t* = 3.05, *p* = 0.004). Thus, the acute change in negative mood was the salient variable which increased tobacco choice, rather than absolute negative mood pre- or post-induction.

**Fig. 3:**
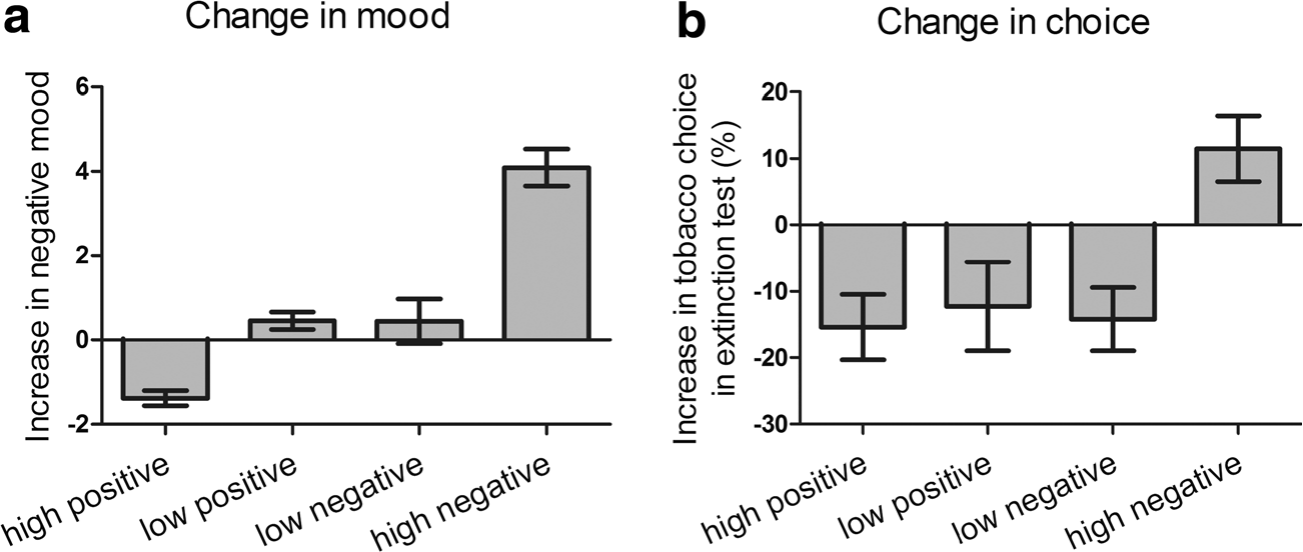
**a** Change in self-reported mood (pre- to post-induction) in four sub-groups. Positive mood values reflect increased sadness, whereas negative values reflect increased happiness. The high-negative group showed a significant increase in sadness, the high-positive group showed a significant increase in happiness, and the two low groups showed no significant change in mood. **b** Change in tobacco choice (from baseline to extinction test with satiety mood induction in between) in the four sub-groups. Positive tobacco choice scores reflect increased tobacco choice, whereas negative scores reflect decreased tobacco choice. The high-negative group showed a significant increase in tobacco choice despite satiety, whereas the remaining three sub-groups all showed a significant decrease in tobacco choice

A standard median split procedure was used to group participants into whether they showed a high or low change in mood consistent with their mood induction procedure, shown in Fig. 3a. ANOVA on these mood change scores (pre- to post-induction) with the factor sub-group (4) yielded a significant main effect, *F*(3.40) = 48.06, *p* < 0.001, *ŋ*_p_^2^ = 0.78. Furthermore, contrasts of pairs using Bonferroni post-hoc tests indicated that the high-negative (*n* = 11) and high-positive (*n* = 13) sub-groups differed from all other sub-groups, *ps* ≤ 0.003, whereas the low-negative (*n* = 9) and low-positive (*n* = 11) sub-groups did not differ from each other (*p* = 1). Finally, the high-negative sub-group’s mood change was significantly greater than zero, *t*(10) = 9.38, *p* < 0.001, and the high-positive sub-group’s mood change was significantly less than zero, *t*(12) = −7.67, *p* < 0.001, whereas the low-negative and low-positive sub-groups combined showed no significant change in self-reported mood, *t*(19) = 1.76, *p* = 0.09.

The important analysis concerns change in tobacco choice by the four sub-groups, the mean and SEM for which is shown in Fig. 3b. ANOVA on tobacco choice data with the variable sub-group (4) yielded a significant main effect, *F*(3.40) = 5.54, *p* = 0.003, *ŋ*_p_^2^ = 0.29. Furthermore, contrasts of pairs using Bonferroni post-hoc tests indicated that the high-negative sub-group differed from all other sub-groups, *ps* ≤ 0.02, whereas the remaining three sub-groups did not differ from each other (*ps* = 1.0). Within-groups *t* tests indicated that whereas the high-negative sub-group showed a significant increase in tobacco choice at test, *t*(10) = 2.32, *p* = 0.04, the remaining three sub-groups combined showed a significant decrease in tobacco choice at test, *t*(32) = −4.47, *p* < 0.001. These within-group *t* tests were significant even when the Holm–Bonferroni correction was applied. Finally, there was no reliable difference between the four sub-groups with respect to baseline choice, *F* < 1, nor when baseline choice was contrasted between the high- and low-negative group, *F* < 1, or high- and low-positive group, *F* < 1, indicating comparability of tobacco choice at baseline. Thus, a significant increase in self-reported negative mood augmented tobacco choice opposing satiety, whereas variations in self-reported positive mood had no impact on tobacco choice.

Importantly, the two negative sub-groups were not confounded with respect to their experience of the devaluation treatment—they showed no reliable difference in breath CO, number of puffs consumed, pleasure of puffs consumed or desire for tobacco, *Fs* < 1—nor were they confounded with respect to participant characteristics—they showed no reliable difference in age, cigarettes smoked per day, years smoking, age of smoking onset, CDS-5 or QSU scores, *ts*(18) < 1.2, *ps* > 0.23. Thus, increased self-reported negative mood was the crucial factor increasing goal-directed tobacco choice, opposing satiety.

## Discussion

The study of young adult daily smokers found that smoking satiety decreased tobacco-seeking in the extinction test of the outcome-devaluation procedure, compared with the abstinent baseline, replicating previous studies (Hogarth 2012; Hogarth and Chase 2011; Hogarth et al. 2013). This devaluation effect indicates that tobacco choice is goal directed in being determined by knowledge of the response-outcome contingencies acquired in concurrent training combined with knowledge of the low value of the tobacco outcome in the sated state (incentive learning). The novel finding of the current study was that this devaluation effect on goal-directed tobacco-seeking produced by satiety was abolished by negative mood induction (compared with positive mood induction). This abolition of the devaluation effect shown in Fig. 1 is ambiguous in its interpretation. This effect could be produced by negative mood impairing retrieval of goal-directed knowledge at test, resulting in no change in responding, as has been found with stress induction (Schwabe and Wolf 2009, 2010), acute alcohol (Hogarth et al. 2012) and alcohol expectancy (Hogarth et al. 2013). Alternatively, the abolition of the devaluation effect in Fig. 1 could be produced by negative mood (relative to positive mood) functioning as a motivational state, raising the expected value of tobacco opposing satiety in the control of goal-directed tobacco-seeking (c.f. Willner and Jones 1996). According to the incentive learning interpretation, negative mood has acquired similar incentive properties to drug withdrawal, which has been shown to raise the expected value of the drug controlling drug-seeking in a direction opposite to satiety (Hutcheson et al. 2001). The incentive learning interpretation also accords with other animal studies which have shown that if internal states which predict opposite values of the reinforcer are manipulated simultaneously, they produce opposing effects on goal-directed action selection in the extinction test similar to that found here (Balleine et al. 1994; Balleine and Dickinson 1994; see also, DeGrandpre et al. 1992; Troisi et al. 2012; Weiss 1972; White and Stolerman 1996).

The sub-group analysis distinguishes between the impaired goal-directed control and the incentive learning accounts of Fig. 1. The sub-group analysis of Fig. 3 showed that participants who reported a significant increase in negative mood significantly increase their tobacco choice in the extinction test, whereas all other participants significantly decreased their tobacco choice in the extinction test. This finding cannot be explained by negative mood impairing goal-directed control because this process would produce no change in choice at test. By contrast, the increase in tobacco choice in the high-negative group can be explained by negative mood functioning as a motivational state raising goal-directed tobacco-seeking in opposition to satiety.

The post-hoc nature of the assignment of participants to the sub-groups means that a range of confounding factors could explain the increase in tobacco choice in the high-negative group other than the increase in negative mood per se. Several potential confounds can be excluded however. Multiple regression analysis indicated that the increase in goal-directed tobacco-seeking was uniquely predicted by the increase in negative mood, rather than absolute negative mood either before or after induction. In addition, the sub-groups were comparable in their baseline tobacco choice, demographic variables and experience of the devaluation treatment, suggesting these variables were not responsible for the increase goal-directed tobacco choice. Finally, the two positive sub-groups did not differ in their change in tobacco choice, so one might conclude that it was negative mood specifically, rather than general arousal produced by the induction protocol, which increased goal-directed tobacco choice at test. However, we did not measure the general arousal induced in each sub-group, so it remains possible that greater arousal, rather than negative mood per se, drove the increase in tobacco choice in the high-negative group (perhaps akin to a stress induction procedure). This issue remains to be explored. Finally, a range of unmeasured individual differences such as depression symptoms (Audrain-McGovern et al. 2014) might have been confounded with the sub-group assignment and driven the increase in tobacco choice at test. Future studies should seek to fully characterise individual differences in sensitivity to negative mood induced priming of goal-directed drug-seeking.

Theories which claim that negative mood primes tobacco-seeking via direct, automatic, unconscious, habitual or compulsive associations, including negative reinforcement, allostasis and incentive habit theory (Baker et al. 2004; Belin et al. 2013; Koob 2009; Koob and Volkow 2010), might seek to defend their position by attributing the current effects to the formation of S-R associations within the task. There are three principled objections to this claim. First, on the S-R account, changes in responding can only be brought about by direct experience of the outcome modifying the S-R association controlling the response (Dickinson 1985; Dickinson and Balleine 2010). As outcomes were omitted in the extinction test, the change in responding produced by satiety and negative mood could not be driven by changes in S-R strength driven by a change in the direct experience of the tobacco outcome, but must have been mediated by an expectation of the tobacco outcome. Second, negative mood was induced between baseline concurrent training and the extinction test, such that the tobacco-seeking response was never reinforced in the presence of the negative mood state. Consequently, negative mood could not have formed an S-R association with the tobacco-seeking response to control performance at test. Third, if S-R associations have formed between negative mood and smoking behaviour in the natural environment, such learning could only *generalise* to promote tobacco over chocolate-seeking at test via a representation of the reinforcer shared between these specific responses, and this proposal is beyond the scope of S-R theory.

One final defence of S-R theories would be to suggest that the current evidence that negative mood functions as a motivational state raising the expected value of drug reinforcement does not exclude the possibility that negative mood might also undergo S-R learning with respect to responses which have been directly reinforced in the mood state. Moreover, such S-R based control by the negative mood state over drug-seeking might be more pronounced in more dependent or psychiatry ill drug users, accounting for their transition to clinical drug dependence. Consistent with this view, there is some evidence that smoking behaviour is more prone to habitual control, indexed by its null correlation with subjective craving, compared with a novel tobacco-seeking response, in more impulsive smokers (Hogarth 2011; see also, Gass et al. 2014). Despite this, the burden of proof currently rests on habit theorists to provide positive evidence that negative mood can acquire S-R control over drug-seeking in ‘ecologically valid’ schedules that mimic complex human learning environments (Kosaki and Dickinson 2010; Sjoerds et al. 2014).

A difficult question remains as to how negative mood becomes established as a signal for the greater reinforcement value of smoking, because there is little evidence that negative mood induction actually increases the liking of smoking (Perkins et al. 2010a); although stress induction does increase smoking liking (McKee et al. 2011; Zinser et al. 1992) and studies are somewhat equivocal as to whether depression prone smokers report increases in smoking liking (Audrain-McGovern et al. 2014; Perkins et al. 2010b). Furthermore, even though smokers who report depressed mood claim to smoke to alleviate depression (Lerman et al. 1996), there is also little evidence that smoking alleviates either experimentally induced negative mood (Conklin and Perkins 2005; Kassel and Shiffman 1997; Kassel and Unrod 2000; Perkins et al. 2008, 2010a; Willner and Jones 1996), or depression (Colman et al. 2011). On the contrary, long-term abstinence appears to improve depression (Cavazos-Rehg et al. 2014; Mathew et al. 2013; Piper et al. 2013; Prochaska et al. 2008; Shahab et al. 2013). Therefore, in subjective report at least, there is little indication that negative mood and depression are associated with the greater reward value of smoking.

This problem for the self-medication hypothesis has been answered in at least two ways (Kassel et al. 2003; Khantzian 1997). One answer is that induced negative mood and depression mimic withdrawal-related negative mood (Baker et al. 2004; Heilig et al. 2010; Hughes 2007; Parrott 1999), which is reliably alleviated by smoking (Hatsukami et al. 1984; Hughes et al. 1984; Parrott 1995; Perkins et al. 2010a). On this view, negative mood is a partially reliable predictor of the greater reward value of smoking. One objection to this claim is that if negative mood was a partial predictor, it should not have competed so effectively against the more reliable predictor, satiety, for the control over tobacco-seeking (White and Stolerman 1996).

Another possibility is that the correction of anhedonia (engagement with reinforcers) by smoking, rather than correction of negative mood per se, provides the additional reinforcement signal. Support for this claim comes from the finding that anhedonia increases during abstinence and predicts relapse (Goelz et al. 2014; Leventhal et al. 2008, 2014a) and can be corrected by acute smoking or nicotine (Dawkins et al. 2006; Donny et al. 2003; Liverant et al. 2014; Pergadia et al. 2014; Perkins and Karelitz 2013; Powell et al. 2002). On this view, smoking is more reinforcing in the negative mood state because it alleviates co-occurring anhedonia, increasing engagement with natural rewards (Ahmed and Koob 2005) rather than ameliorating negative mood per se. As anhedonia increases with the transition clinical drug dependence (Koob 2013), and withdrawal-related anhedonia is more pronounced in depressed individuals (Pergadia et al. 2014), negative mood should become a more powerful motivational state, raising the expected value of the drug, as dependence grows. Thus, incentive learning driving supernormal goal-directed drug choice to acutely correct burgeoning anhedonia could be the learning mechanism that underpins the transition to clinical drug dependence, consistent with choice based theories (Bickel et al. 2014; Henden et al. 2013; Heyman 2013) over automaticity theories of dependence (Belin et al. 2013; Everitt and Robbins 2013; Koob 2013; Koob and Volkow 2010; Tiffany 1990).

As depression and anhedonia are associated with reduced learning about natural reward contingencies in smokers (Liverant et al. 2014; Pergadia et al. 2014), it is possible that negative mood simultaneously reduced the value of chocolate and increased the value of smoking, expanding the differential value between these two outcomes, driving up the proportion of tobacco choices (this could explain why the high-negative group chose tobacco above their abstinent baseline). The concurrent choice procedure means that we cannot rule out the possibility that negative mood reduced chocolate value. However, there is substantial evidence that negative mood augments the value of smoking when tested independently of a natural reward alternative (Brandon et al. 1996; Conklin and Perkins 2005; Fucito et al. 2010; Payne et al. 1991; Perkins et al. 2013; Vinci et al. 2012; Willner and Jones 1996), so we can be confident in this aspect of the interpretation. By contrast, Conklin and Perkins (2005) found no change in motivation for water following negative mood induction, suggesting that negative mood does not reduce natural reward value. Furthermore, depression is prospectively linked to developing obesity, suggesting that negative mood may not undermine food motivation (Luppino et al. 2010). Nevertheless, the possibility that negative mood changes the value of both two outcomes, to bias action selection, remains an important consideration that requires direct testing.

One remaining question concerns the relevance of the current findings to depression. Depression is a heterogenous disorder made up of a cluster of symptoms including negative mood, anhedonia, cognitive impairment, concentration difficulty, neurovegetion, psychomotor retardation, appetite loss and insomnia. It is unlikely that the mood induction procedure used in the current study impacted on depressive symptoms outside negative mood, particularly the non-affective symptoms. Therefore, the study cannot speak about the role these other symptoms play in the maintenance of drug use. The other issue is that major depression is more severe and chronic than the transitory induced negative mood state. A diagnosis of depression requires that depression lasts for at least 2 weeks and is associated with a clinically significant change in functioning. Thus, there are both qualitative and quantitative differences between mood induction and depression. This analysis does not discount the importance of the present findings. Indeed, the present findings may have more broad spanning implications beyond depression, addressing how negative affect modulates expected outcome values in healthy people and across those with different clinical diagnoses, including depression, anxiety and psychoses, all of which have an affective component. Finally, our major claim that emergent, abnormal incentive learning underpins the transition to drug dependence is undermined by young health smoker cohort studied here. Extensive testing in clinical samples is requires before this claim becomes anything more than a plausible possibility.

To conclude, numerous studies have shown that negative mood induction increases drug motivation, but it was previously unclear if this effect could compete with primary motivational states. Our unique finding that negative mood fully countered satiety demonstrates that negative mood is a strong motivational state, capable of competing with primary motivational states. Furthermore, negative mood increased drug-seeking in an extinction test suggesting that negative mood, relative to positive mood, functions as a motivational state as envisaged by incentive learning theory, raising the expected value of the drug to drive goal-directed drug-seeking. This finding contradicts S-R theories which claim that negative mood controls drug-seeking directly. More speculatively, the data might suggest that psychiatric illness writ large confers vulnerability to drug dependence not by promoting automatic control of behaviour but by enabling incentive learning, wherein psychiatric states drive supernormal goal-directed drug choice in order to acutely correct (but ultimately exacerbate) those psychiatric states.
